# Impaired Hair Growth and Wound Healing in Mice Lacking Thyroid Hormone Receptors

**DOI:** 10.1371/journal.pone.0108137

**Published:** 2014-09-25

**Authors:** Constanza Contreras-Jurado, Laura García-Serrano, Mónica Martínez-Fernández, Lidia Ruiz-Llorente, Jesus M. Paramio, Ana Aranda

**Affiliations:** 1 Department of Physiopathology of the Endocrine and Nervous Systems, Instituto de Investigaciones Biomédicas “Alberto Sols”, Consejo Superior de Investigaciones Científicas and Universidad Autónoma de Madrid, Madrid, Spain; 2 Molecular Oncology Unit, Division of Biomedicine, CIEMAT, Madrid, Spain; University Claude Bernard Lyon 1, France

## Abstract

Both clinical and experimental observations show that the skin is affected by the thyroidal status. In hypothyroid patients the epidermis is thin and alopecia is common, indicating that thyroidal status might influence not only skin proliferation but also hair growth. We demonstrate here that the thyroid hormone receptors (TRs) mediate these effects of the thyroid hormones on the skin. Mice lacking TRα1 and TRβ (the main thyroid hormone binding isoforms) display impaired hair cycling associated to a decrease in follicular hair cell proliferation. This was also observed in hypothyroid mice, indicating the important role of the hormone-bound receptors in hair growth. In contrast, the individual deletion of either TRα1 or TRβ did not impair hair cycling, revealing an overlapping or compensatory role of the receptors in follicular cell proliferation. In support of the role of the receptors in hair growth, TRα1/TRβ-deficient mice developed alopecia after serial depilation. These mice also presented a wound-healing defect, with retarded re-epithelialization and wound gaping, associated to impaired keratinocyte proliferation. These results reinforce the idea that the thyroid hormone nuclear receptors play an important role on skin homeostasis and suggest that they could be targets for the treatment of cutaneous pathologies.

## Introduction

It is known that the skin is a target organ for thyroid hormones and that changes in thyroidal status can lead to skin alterations [1]–[3]. In fact, for more than one century hypothyroidism has been known as mixedema, referring to the skin edema observed in hypothyroid patients. In these patients the epidermis is thin and dry and alopecia is common [4], indicating that thyroidal status might influence not only skin proliferation but also hair growth. [5]–[8]. Hair undergoes periods of degeneration (catagen), rest (telogen) and growth (anagen), generating and maintaining the hair cycling. An intricate network of signals that affect multiple developmental pathways, such as Wnt, BMP, Hedgehog and Notch [9]–[11], which modulate specific transcriptional programs, mediates these changes. It has been described that the thyroid hormones induce anagen prolongation and hair matrix keratinocyte proliferation [8]. Furthermore, hypothyroid patients under thyroid hormone treatment sometimes report hair growth as a *side effect*, and alopecia has been found in patients with resistance to thyroid hormone (RTH syndrome) [12]. Further supporting a role for thyroid hormones on hair growth, topical application of a thyroid hormone analog was found to be efficacious in both mouse and monkey models [13] and also stimulates epidermal proliferation and hair growth in murine models [14].

Wound healing is a complex tissue remodeling process in which the damaged tissue is removed and substituted with normal skin [15], [16]. This process involves proliferation of keratinocytes at the wound edges and their migration, as well as the formation of a granulation tissue that will originate a mature dermal tissue [17]. The thyroid hormones can also play a role in wound healing [18]. Topical treatment with thyroid hormone accelerates wound healing in rodents [19], [20], and it could also be effective in humans [21]–[23].

Most actions of the thyroid hormones are mediated by binding to nuclear receptors (TRs) that belong to the superfamily of nuclear receptors and act as ligand dependent transcription factors. TRs are encoded by two different genes, and TRα1 and TRβ are the main thyroid hormones binding isoforms [24]. We recently found that in KO mice lacking TRs there is reduced keratinocyte proliferation and decreased hyperplasia of the interfollicular epidermis in response to topical application of 12-*O*-tetradecanolyphorbal-13-acetate (TPA) or retinoids. We showed that the effects of the thyroid hormones on skin proliferation are mediated through interactions with TRα and TRβ and that both receptor genes contribute to attain a normal proliferative response [25], [26]. In this work we report that TRs disruption in mice also causes a reduction of follicular cell proliferation and anagen entry similar to that found in hypothyroid mice. In addition, TRs deletion retarded wound healing, decreasing the re-epithelialization process, further showing that thyroid hormone actions on the skin require binding to their nuclear receptors.

## Results

### Follicular proliferation and anagen entry is impaired in TR-deficient mice

**Figure 1:**
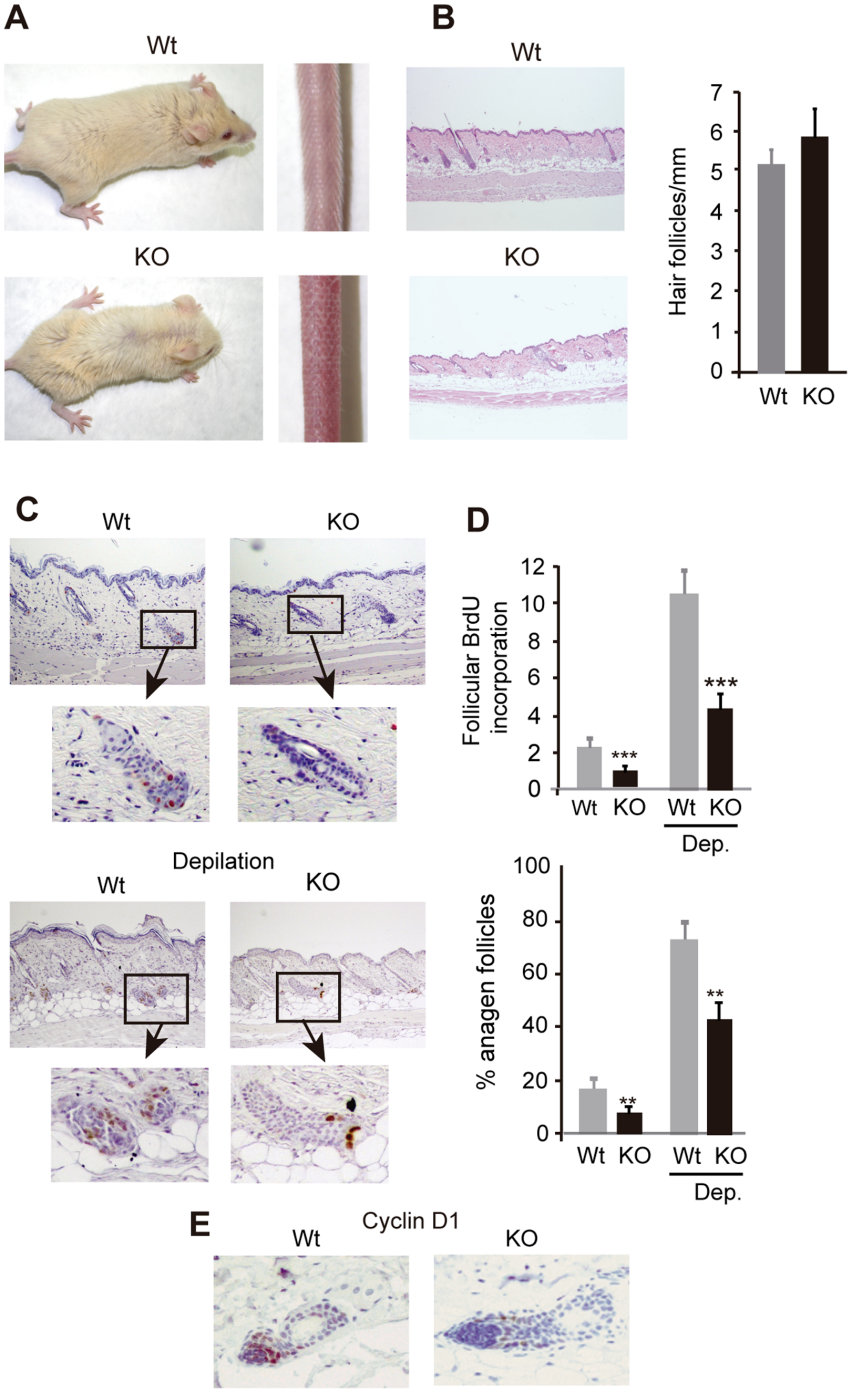
Influence of TRs on hair growth. (**A**) Skin phenotype of wild-type (Wt) and TRα1^−/−/^TRβ^−/−^ KO mice. (**B**) Immunohistochemical images of follicular BrdU incorporation in the dorsal skin of untreated Wt and TR KO mice and in mice depilated five days before. Larger magnifications of the hair follicles are also shown. (**C**) Proliferation rate of the follicular keratinocytes was quantified and is represented as the % of positive BrdU cells vs. total hair follicle cells. Percentage of hair follicles in anagen is shown in the lower panel. Data are shown as mean values ± SE, and asterisks denote statistically significant differences relative to Wt mice (*, P<0.05; **, P<0.05***, P<0.001). (**D**) Representative immunohistochemistry showing follicular expression of Cyclin D1 in depilated animals of both genotypes.

**Figure 2:**
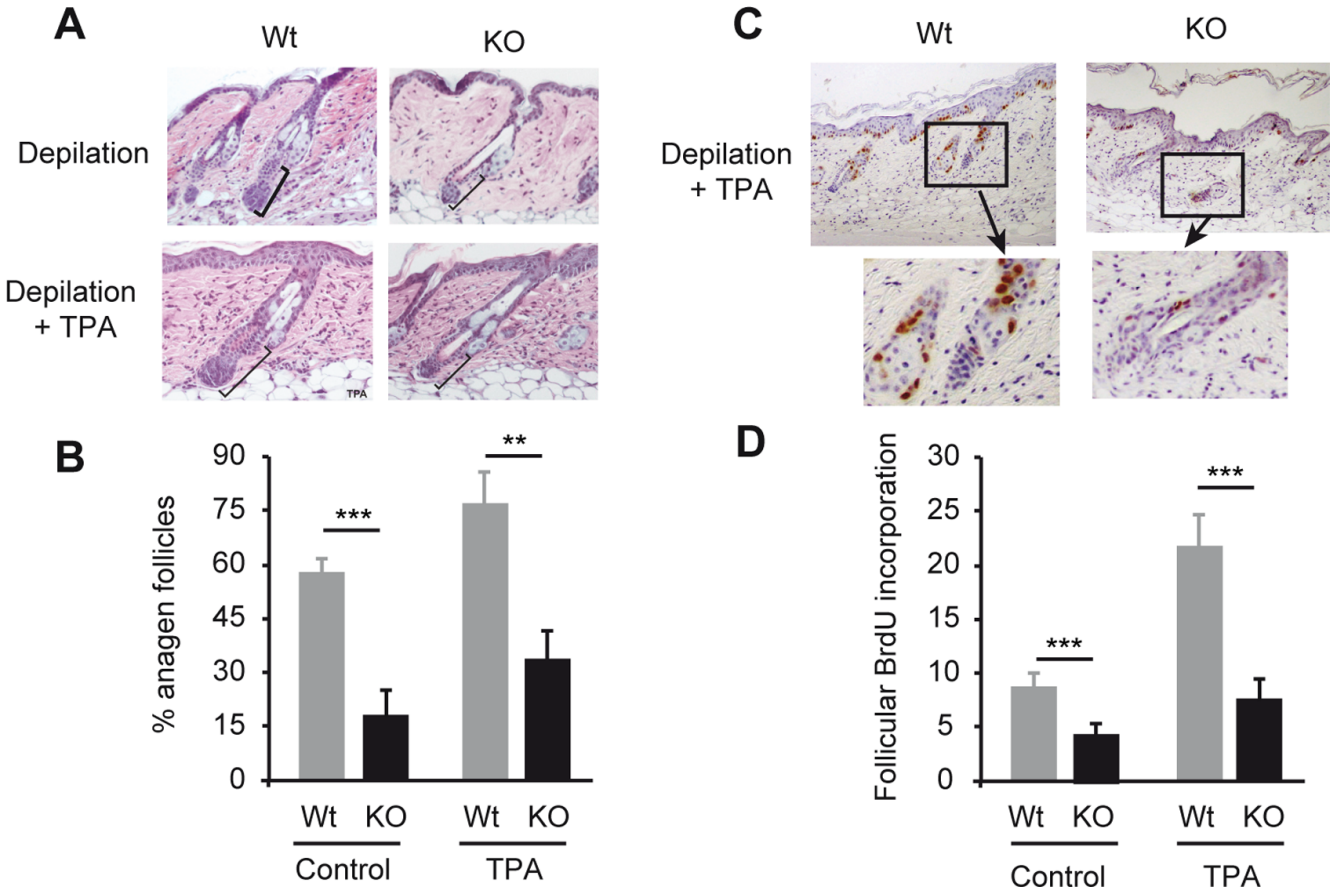
Reduced hair follicle response to TPA in TR-deficient mice. (**A**) Representative H&E images of dorsal skin of Wt and TR KO mice that were depilated and then treated topically with vehicle (acetone) or TPA. The distance between the dermal papilla and the sebaceous gland is indicated with black brackets. (**B**) Quantification of the % of follicles in anagen phase in the different conditions. (**C**) Immunostaining of BrdU in the TPA-treated mice. The lower panels show amplification of the follicles indicated in the upper panel. (**D**) Follicular BrdU incorporation represented as the percentage of positive BrdU cells *vs.* total hair follicle cells before in vehicle and TPA-treated animals.

With age mice lacking TRs show an obvious hair phenotype, with a less thick fur and occasional hair tail loss (Fig. 1A). Nonetheless, the number of hair follicles was similar in Wt and TR-deficient mice, indicating that these receptors are not required for follicle development and/or maintenance (Fig. 1B). We next analyzed whether TRs could influence hair follicle proliferation. As shown in Fig. 1C, D, follicular BrdU incorporation was markedly reduced in TR KO mice with respect to Wt animals under resting conditions. In addition, a significantly lower number of follicles in anagen, the growing phase of the hair cycle, were observed in the TR-deficient mice (Fig. 1D). To analyze anagen responses in these mice, we first stimulated hair growth by exhaustive depilation [27]. This treatment induced a stronger anagen entry in Wt than in KO mice. Coincidentally, the proliferation index in the hair follicles of the TR KO mice was significantly lower than in Wt. follicles (Fig. 1C, D). In addition, a reduced expression of Cyclin D1, a marker of follicular proliferation was also observed (Fig. 1E). Furthermore, we analyzed the response to TPA that causes anagen entry [28]. In contrast to Wt mice, follicular proliferation and hair follicle length were not significantly increased in TR KO mice that were treated with TPA after depilation, reflecting a defective anagen response in these mice also after the treatment with this hyperproliferative agent (Fig. 2).

**Figure 3:**
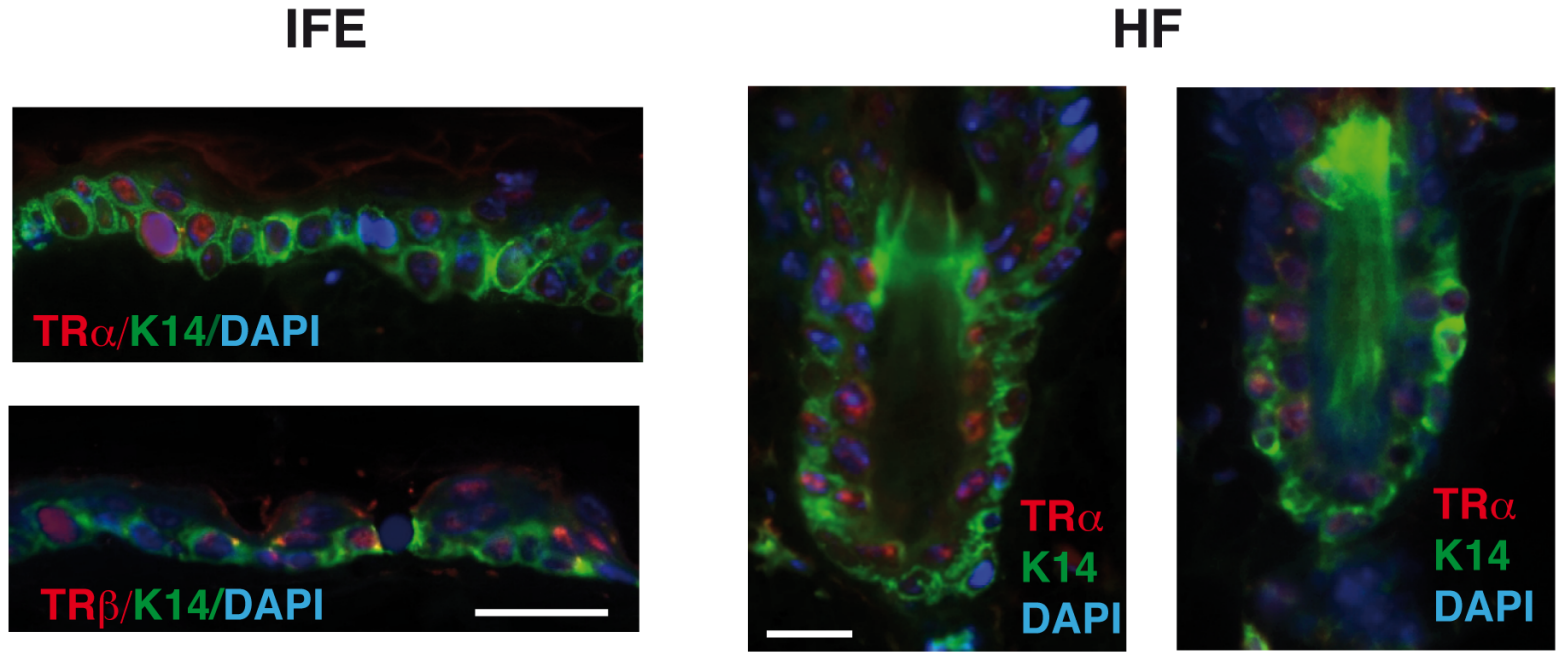
Both TRα and TRβ are expressed in the mouse skin. Double immunofluorescence images of expression of keratin 14 (K14, green) with TRα or TRβ (red). Images were obtained from the interfollicular epidermis (IFE) and the hair follicles (HF) of wild-type mice. The slides were counterstained with DAPI (blue) and the merged images are shown. Bars: 50 µM.

**Figure 4:**
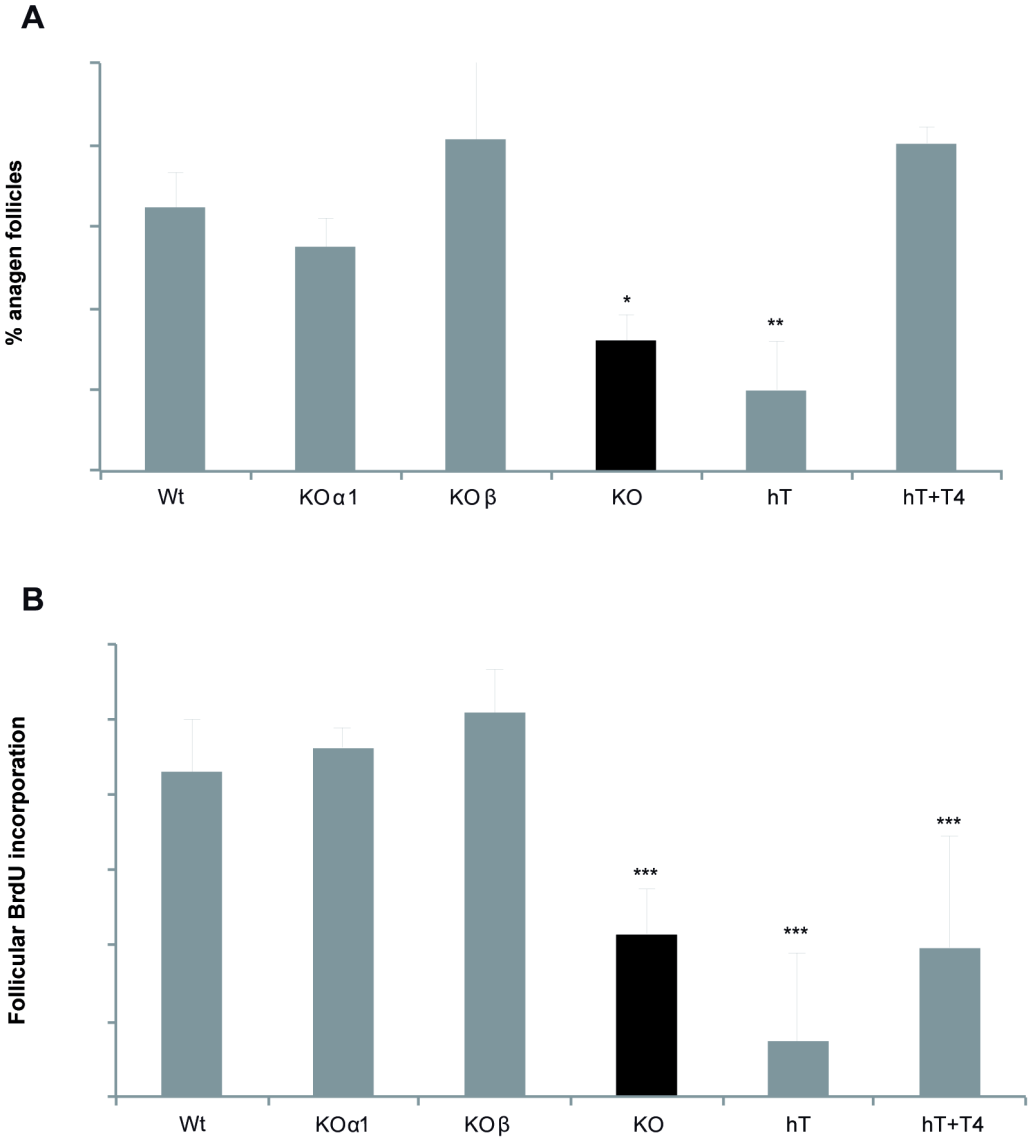
Deletion of both TRα1 and TRβ is required to impair follicular proliferation and the anagen response to depilation. (**A**) Percentage of follicles in anagen growing phase and (**B**) Follicular BrdU incorporation, 5 days after exhaustive depilation. Experiments were assed in Wt animals, animals lacking both TRα1 and TRβ (KO), and in the individual KOs for TRα1 and TRβ (KOα1 and KOβ, respectively). Data are shown as mean values ± SE and the asterisk denotes a statistically significant difference (P<0.05) of KO vs Wt mice. Defective follicular proliferation and anagen response to depilation in hypothyroid mice. The percentage of hair follicles in anagen and BrdU incorporation in the follicular keratinocytes was quantified 5 days after depilation in Wt control mice (Ct), in mice made hypothyroid by treatment with antithyroidal drugs for 4 months (hT) and in a group of animals receiving the anti-thyroidal drugs plus thyroxine (hT+T4). A group of age-matched double KO mice were also used. Data are represented as the % of positive BrdU cells vs. total hair follicle cells. Data are shown as mean values ± SE, and asterisks denote statistically significant differences vs Ct mice. *, P<0.05; **, P<0.01.

To examine the contribution of the TRα and TRβ receptors to hair growth, we first analyzed by immunohistochemistry their expression in the skin of Wt mice. As shown in Fig. 3, both receptor isoforms were expressed not only in the interfollicular epidermis but also in the hair follicles. As both receptors were present, we next compared the response to depilation of the double KO mice with that of single KO mice deficient in either TRα1 or TRβ. In contrast with the results obtained in mice lacking both receptor isoforms, single KO mice did not show impaired follicular proliferation or anagen entry (Fig. 4), supporting that both receptors participate in this response.

To analyze the role of the ligand on hair growth, we also conducted experiments in hypothyroid mice. As shown in Fig. 4, the results obtained were similar to those observed in mice lacking the receptors, since both follicular BrdU incorporation and anagen entry after depilation were impaired in hypothyroid mice. Furthermore, these defects were reversed when mice were treated daily with a physiological dose of thyroid hormone.

**Figure 5:**
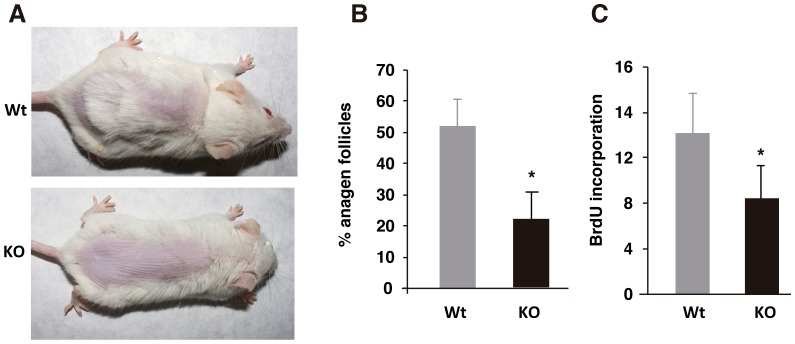
TR-deficient mice develop alopecia after serial depilation. (**A**) Wt and TR KO mice were depilated once a week and pictures were taken after 21 weeks. Quantification of anagen follicles (**B**) and follicular BrdU incorporation (**C**) in both groups at the end of the experiment. Data are shown as mean ± SE. *, *P*<0.05.

To further analyze the role of TRs in hair growth, we next conducted a chronic experiment in which animals were depilated once a week for 21 weeks (Fig. 5). In Wt animals, hair growth was observed during the whole experimental period. However, in TR KO mice there was a clear retardation in the induction of hair growth and in the last weeks they developed total alopecia or presented hair re-growth in very small isolated areas. At sacrifice, a significant decrease in the percentage of anagens and follicular BrdU incorporation was observed, indicating again the important role of TRs on hair growth.

### Wound healing is retarded in TR-deficient mice

**Figure 6:**
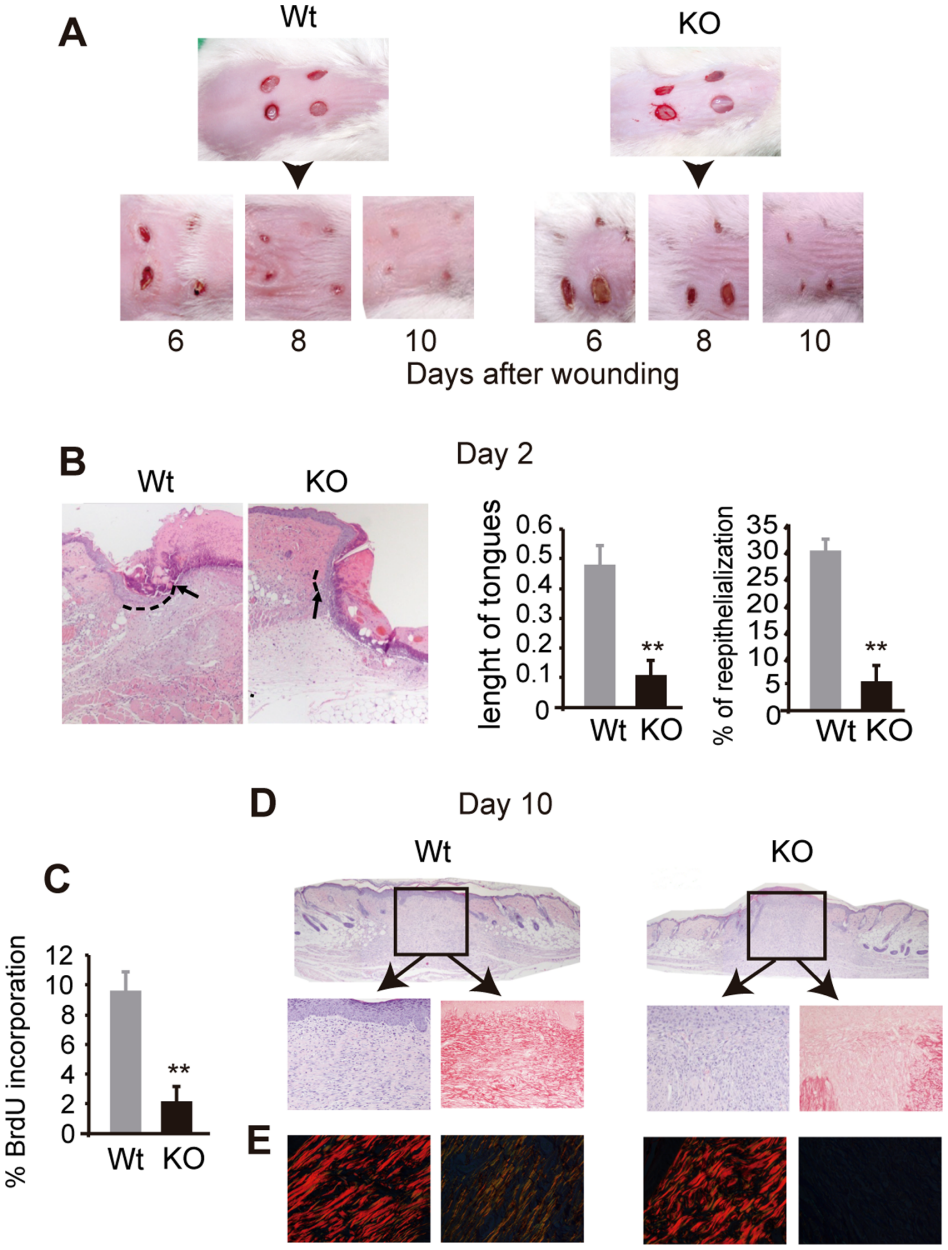
Wound healing is retarded in mice lacking TRs. (**A**) Representative examples of wound closure in control and TR KO mice. Images were taken at the indicated times after wounding. (**B**) H&E staining 2 days after wounding. The black arrows indicate the end of the keratinocyte tongues. The main length of the tongues and the % of wound re-epithelialization are shown in the right panels. (**C**) 10 days after wounding BrdU positive cells were quantitated from histological sections at the wound edges in Wt and KO mice. Data are means ± SE. **, *P*<0.01. (**D**) H&E staining of wounds at 10 days. The lower panels show a detail of H&E and picrosirius staining of the indicated areas. (**E**) Collagen fibers detected under polarized light of picrosirius staining in the unwounded tissue (left panels) and the wound centers (right panels).

We next analyzed whether wound healing was also altered in the absence of TRs. With this aim, skin wounds were made in Wt and TR-deficient mice and the healing process was followed. A slower rate of healing was apparent in TR KO mice (Fig. 6A), and differences were observed as early as 2 days after wounding (Fig. 6B). During wound healing, migrating keratinocytes form an epithelial tongue of a few undifferentiated cell layers, which establish the leading wound edge. KO mice showed clearly retarded re-epithelialization as judged by the reduced length of the epithelial migrating tongue, and by the smaller percentage of re-epithelized wound (Fig. 6B).

After wounding, a rapid proliferation of the epithelial cells immediately next to the migrating epithelial tongue is observed. At 10 days after wounding, epidermal proliferation was significantly lower in the epidermis of TR-deficient mice, as reflected by the reduced number of BrdU-positive keratinocytes in the transitional compartment adjacent to the wound edge (Fig. 6C). In addition, histological analysis showed that hyperplasia was reduced and the basal lamina was not still well defined in these animals, indicating again a strongly retarded wound healing (Fig. 6D). The rate of re-epithelialization usually links to the maturation of the dermal tissue [16], and the granulation tissue (a provisional tissue that will originate the new dermis), was more mature and abundant in the wounds of the wild type mice than in those of the mice lacking TRs (Fig. 6B and D). This was further evidenced by Picrosirius staining of day 10 wounds, which showed a clear retardation of wound collagen deposition in granulation tissues of TR KO vs. Wt mice. When staining of the wound center was observed under polarized light, the presence of collagen fibers at different maturation states could be detected in the Wt mice but not in the TR KO mice. This demonstrates that TRs are also important for regulating collagen deposition in fibroblasts during wound healing (Fig. 5E).

### Keratinocytes from TR KO mice present enhanced migratory capacity

**Figure 7:**
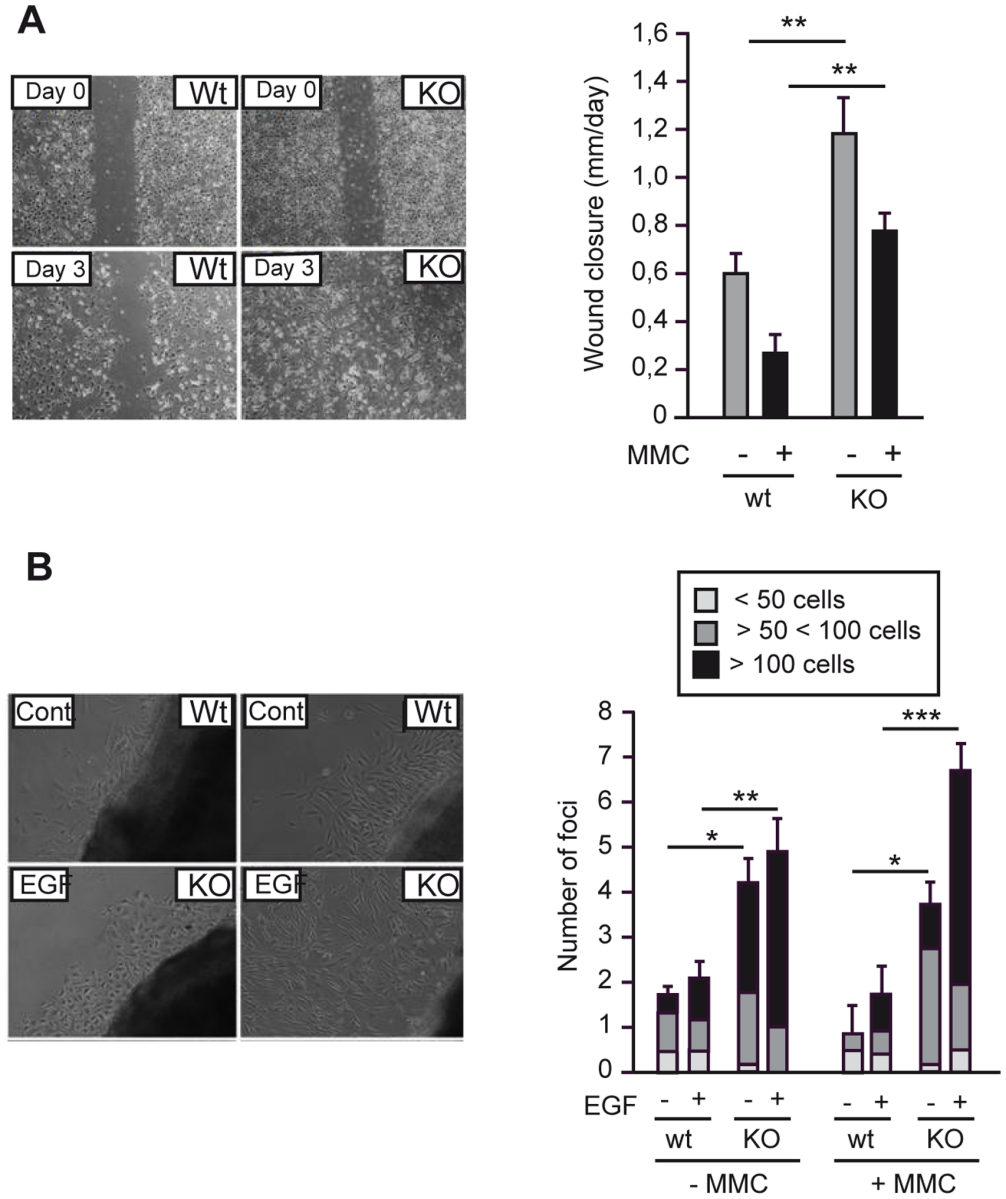
Migration in vitro is enhanced in keratinocytes obtained from TRs deficient mice. (**A**) Representative images of scratch wound assays in primary cultures of newborn keratinocytes from Wt and TR KO mice. Confluent cultures were treated with mitomycin C (MMC) for 2 h before wounds were made. The right panel illustrates the rate of wound closure obtained in untreated and MMC treated cultures in both genotypes. (**B**) Representative images of dorsal skin explants from newborn Wt and TR KO mice treated with MMC and then incubated during 6 days in the presence and absence (Cont.) of EGF. In the right panel the number and size of keratinocyte foci migrating out of the explants in the presence and absence of MMC and EGF were quantitated. Data are shown as mean values ± SD, and asterisks denote statistically significant differences of Wt vs KO cultures. *, P<0.05; **, P<0.01; ***, P<0.001.

The healing process involves both hyperproliferation of keratinocytes in the wound borders that feed the epithelial tongue, as well as their migration until wound closure is attained [17]. In primary cultures, we have shown that proliferation of keratinocytes from TR KO mice is intrinsically reduced when compared with that of Wt animals [25]. We now asked whether a reduced keratinocytes migratory capacity could also be involved in the delayed healing phenotype in TR KO mice. As shown in Fig. 7A, reduced migration in TR-deficient mice was not found in primary keratinocyte cultures. Rather, we observed that TRs ablation accelerated significantly keratinocyte migration in a scratch wound assay. The observed effects are proliferation independent, because enhanced closure was also observed in TR KO cultures previously treated with mitomycin C (MMC). Similar results were obtained with skin explants. As illustrated in Fig. 7B, the number and size of keratinocyte foci migrating from the explants was significantly increased in KO mice, both in the presence and absence of MMC. Enhanced migration of TR KO keratinocytes was also observed after addition of EGF, a growth and pro-migratory factor [29], which increased migration from the Wt and KO explants.

## Discussion

Both clinical and experimental data indicate that thyroid hormones have a stimulatory effect on hair growth [4]–[8], [14]. In this work we show that disruption of TRα and TRβ, the main receptor isoforms that bind the thyroid hormone, reduces BrdU incorporation in the keratinocytes of the hair follicles and the number of anagens. Although TR-deficient mice show increased follicular proliferation and anagen follicles in response to depilation or to topical administration of TPA, two important stimuli for anagen entry [27], [28], they do not reach the levels attained in the Wt mice. The significant role of the receptors on hair growth is also shown by the finding that TR deficient mice develop alopecia after serial depilation. Cyclin D1 plays an important role in keratinocyte proliferation [30], and we have reported that the decreased interfollicular proliferation found in the TR KO mice was associated to decreased expression of Cyclin D1 [25]. On the other hand, a thyroid hormone analog stimulates epidermal thickening in mice and promotes human keratinocyte proliferation by activating Cyclin D1 expression and promoting entry into the S phase of the cell cycle [31]. Cyclin D1 expression was also reduced in the hair follicles of the TR KO animals and therefore could be involved in the proliferative hair defect observed.

Analysis of the phenotypes of mice in which TRα1 or TRβ have been individually inactivated [32], [33], as well as the distinct syndromes originated in humans by TRα and TRβ mutations [34], show that both receptors can substitute for each other to mediate some actions of the thyroid hormones and that they also have isoform-specific functions. Both TRα and TRβ mRNAs are present in the skin [3], [5], [6], [35]. Although TRβ appears to be the predominant isoform expressed in the human hair follicle, we show evidence that both receptors are expressed in mouse hair follicles and we found that deletion of both TRα and TRβ was required to impair follicular proliferation or anagen entry in mice. This indicates that both receptors have an overlapping or compensatory role in hair cycling in these animals.

Interestingly, a point mutation of the TRα gene with a proline to histidine substitution (P398H) is sufficient to affect hair growth. Mice with this mutation frequently presented delayed hair growth and, in some cases, the mice had no body hair for up to eight weeks of age [36]. This could be due to the strong dominant-negative activity of the TRα mutant receptor, which also causes important metabolic alterations.

The phenotype of mice devoid of TRs is not always identical to that found in hypothyroid mice, since the unliganded receptors can act as repressors of gene expression [37], [38]. However, the defect in hair proliferation found in TR-deficient mice was similar to that observed in mice treated with anti-thyroidal drugs. Furthermore, thyroid hormone administration reversed the reduced proliferation. This shows that the deficiencies observed in the hypothyroid mice are not due to non-specific effects of the anti-thyroidal drugs, but are rather a direct consequence of the altered thyroidal status. Taken together these results demonstrate that binding of thyroid hormones to their nuclear receptors mediates their endocrine actions on hair growth and that receptor loss is equivalent to hormone deficit in this organ.

Our data also revealed that cutaneous wounds heal more slowly and show reduced collagen deposition in animals lacking TRs. This is in agreement with the finding that thyroid hormone administration accelerates wound healing in mice [20]. Re-epithelialization in TR-deficient mice, an early indicator of wound healing [39], was strongly retarded compared with that in Wt mice. As the observed in vivo delayed healing phenotype in TR KO mice involved substantial reduction of re-epithelialization and wound gaping, a reduced migratory capacity of the keratinocytes could also be involved in the healing defect. To analyze this possibility, we tested *in vitro* their migratory behavior using scratch wound closure assays in primary cultures and keratinocyte migration assays from skin explants. The results obtained demonstrated that the delayed re-epithelialization observed in TR KO mice was not secondary to a decreased keratinocyte migratory capacity, since they rather exhibited increased migration *in vitro*. Because the re-epithelialization rate is determined by both proliferation and migration of keratinocytes, these data suggest that retarded re-epithelialization in the absence of TRs is due to a decrease in keratinocyte proliferation.

The increased *in vitro* migratory capacity of the TR KO keratinocytes, as well as their reduced proliferation obtained in primary cultures [25], indicate that keratinocytes are a direct target of the TRs. The finding that TRs are present in the interfollicular epidermis and in the hair follicles reinforces this idea. However, the impaired hair growth and the wound-healing defect found in mice lacking TRs could also be secondary to systemic effects or to changes in dermal fibroblasts. The specific deletion of individual and combined TRα and TRβ genes in keratinocytes will hopefully clarify the role of the receptors in these processes.

Hair follicle stem cells are responsible for maintaining hair cycling [40]–[42] and they also support the regeneration of the injured epidermis after wounding [43]–[46]. The skin phenotype of TR KO mice with defective response to proliferative stimuli, retarded hair growth and impaired wound healing would be compatible with the existence of a defect in the number or function of the hair follicle stem cells. Future studies directed towards the identification of the cellular mechanisms and genomic changes underlying TRs actions in these cells will be essential to fully understand the role of the thyroid hormones on skin homeostasis.

Regulation of gene expression by nuclear receptors plays an important role on the skin. The important role of the glucocorticoid (GR), androgen (AR), and estrogen (ER) receptors is well known, but non-steroid receptors, which heterodimerize with the retinoid X receptors (RXR) are also involved in skin proliferation, hair growth and wound healing [47]. Among them, the retinoic acid receptors (RAR and RXR) [48]–[50] and the vitamin D receptor (VDR) [51], [52] are important skin modulators and targets for the treatment of cutaneous pathologies. Additional members of this subgroup of heterodimeric receptors including the peroxisome proliferator-activated receptors (PPAR) [53] [54], the farnesoid X receptor (FXR) [55] and the liver X receptors (LXR) [54], [55] are also significant contributors to skin development and homeostasis. These receptors, as well as TRs, modulate cell proliferation, differentiation, wound healing or cutaneous inflammation [56], [57]. Delineation of the molecular mechanisms by which TRs and other nuclear receptors regulate keratinocyte biology and the interaction among different cellular types in the skin could open new avenues for the treatment of cutaneous diseases.

## Materials and Methods

### Ethics Statement

All animal work was done in compliance with the European Community Law (86/609/EEC) and the Spanish law (R.D. 1201/2005), with approval of the Ethics Committee of the Consejo Superior de Investigaciones Científicas (CSIC) were approved by the Subcommittee on Bioethics of the Consejo Superior de Investigaciones Científicas with the approval reference number BFU2011-28058. All efforts were made to minimize suffering. Anesthetics were used as indicated.

### Animals and Treatment

Animals were housed in temperature (22±2°C) and light (12∶12 light-dark cycle; lights on at 7 a.m.) controlled conditions and had free access to food and water. TRα1^−/−/^TRβ^−/−^ double knockout (KO) mice and the corresponding wild-type TRα^+/+^/TRβ^+/+^ (Wt) animals have been described previously [58]. TRα1^−/−/^TRβ^−/−^(KO), TRα1^−/−^TRβ^+/+^ (KOα1), TRα1^+/+^TRβ^−/−^ (KOβ mice and the corresponding Wt animals were also used. Studies were performed in adult female age-matched animals 2–4 months old. Wild-type mice were made hypothyroid (hT) as described [25], [26] by treatment with 0.02% methymazole and 0.1% sodium perchlorate in the drinking water. This treatment reduced serum levels of thyroxine (T4) by approximately 80% [26]. After 7 days of treatment with the anti-thyroidal compounds, a group of animals was treated daily with a substitutive dose of T4 (20 ng/g BW) (hT+T4). To induce anagens, mice were anesthetized with a mixture of 2 mg/kg ketamine (Imalgene 500, Merial) and 100 µg/kg of medetomidine (Domtor, Pfizer, Spain), dorsal skins were shaved and exhaustively depilated with hair removing cream. Animals were sacrificed 5 days later together with controls depilated just before sacrifice. The percentage of anagen follicles *vs*. the total number of follicles was determined from at least 10 slides/animal of each experimental group (a minimal of 8 animals/group). In serial depilation experiments, 4 mice/genotype were depilated once a week during 21 weeks. In order to label proliferating cells in hair follicles, 1 h prior sacrifice mice were injected ip with bromodeoxyuridine (BrdU; 0.1 mg/kg weight).

### Scratching assays

Primary keratinocytes were isolated from newborn KO and wild-type mice as previously described [25], [26]. For migration assays, the cell monolayers were treated for 2 h in the absence or presence of 10 µM mitomycin C (MMC) and scarred with a sterile micropipette tip. Scratch recovering was followed for 3 days and photographs were taken at the beginning of the assay (*t* = 0 h) and every 24 h to calculate distance. Magnification 100×.

### Skin explants

Skin explants were prepared from 2-day-old pups as described previously [59]. A 4-mm sterile punch was used to remove explants, which were placed into 24-well plates containing coverslips. Explants were either untreated or treated with MMC for 2 h and then cultured for 6 days in medium with low CaCl_2_ concentration to avoid fibroblast growth. Explants were cultured with medium alone or in the presence of epidermal growth factor (EGF; 20 ng/ml). To assess keratinocyte outgrowth, phase-contrast micrographs of explants were taken at day 6. Micrographs were scanned and outgrowth was quantitated counting the number and size of keratinocyte foci. Foci were classified into three groups: containing less than 50 cells, containing between 50 and 100 cells and containing more than 100 cells. The vast majority of cells were keratinocytes, as identified by immunodetection with K5.

### In vivo wound healing analysis

Mice were injected intraperitoneally with a mixture of 20 mg/kg ketamine and 100 µg/kg of medetomidine. Once the mice were adequately anesthetized, four 4 mm wounds were punched in the depilated dorsal skin. Animals (3–4/group) were sacrificed 2 and 10 days after wounding. Biopsies containing the wounded tissue and the surrounding skin were harvested, fixed with paraformaldehyde and embedded in paraffin. H&E staining was performed on 4 µm serial sections, measuring every 10 sections the distance between the unwounded dermis. The percentage of re-epithelialization was calculated measuring from the wound center the distance between the unwounded dermis, considering this length as the initial wound. The length of the epithelial tongues migrating from the unwounded skin was also measured, and this value was considered as the re-epithelialized surface.

### Histological Analysis and Immunohistochemistry

Skin sections (4 µm) were stained with H&E or processed for immunohistochemistry or immunofluorescence. For frozen sections (5 µm), fresh skin samples were embedded in OCT (TissueTech) and kept frozen. Picrosirius red staining was performed for detection of collagen fibers [60]. Picrosirius stained slides were observed under polarized light using a Leica DMBL light microscope microscope equipped with a polarizer/analyzer set. Immunohistochemistry was performed using standard protocols on deparaffinized sections [25], [26]. Slides were mounted and analyzed by light microscopy (Leica DM RXA2), and microphotographs of hair follicles were taken. The Cyclin D1 antibody (Neomarkers RM-9104-R7) was used at a 1/100 dilution. BrdU incorporation was detected by immunohistochemistry (Roche, 1170376). The number of BrdU-positive and total follicular cells of dorsal skin was counted in at least 10 areas of 25 mm^2^/animal of each experimental group. The mean percentage of proliferating over total follicular cells was then estimated. Immunofluorescence was carried out on deparaffinized sections. TRα (600-401-A38 Rockland), TRβ (Affinity BioReagents ABR PA1-213A) and keratin 14 (Ab7800, Abcam) antibodies were used at a 1/100 dilution. Slides were incubated with the antibodies at 4°C overnight and then with anti-rabbit-TexasRed (1/500, 711-075-152 Jackson Immunoresearch) or anti-mouse FITC (1/50, 715-095-15 Jackson Immunoresearch) secondary antibodies at room temperature for 1 h. Sections were examined for fluorescence in a Zeiss Axioplan2 microscopy.

### Statistic analysis

Differences between KO and wild-type animals were assessed by ANOVA followed by the Bonferroni correction, or with the *t* test when only two groups were compared. In the figures differences between wild-type and TR KO mice is indicated as *p<0,05; **p<0,01 and *** p<0,001.
